# The Graphene/l-Cysteine/Gold-Modified Electrode for the Differential Pulse Stripping Voltammetry Detection of Trace Levels of Cadmium

**DOI:** 10.3390/mi7060103

**Published:** 2016-06-13

**Authors:** Yu Song, Chao Bian, Jianhua Tong, Yang Li, Shanghong Xia

**Affiliations:** 1 State Key Laboratory of Transducer Technology, Institute of Electronics Chinese Academy of Sciences, Beijing 100190, China; songyu1990@126.com (Y.S.); jhtong@mail.ie.ac.cn (J.T.); yangli@mail.ie.ac.cn (Y.L.); 2 University of Chinese Academy of Sciences, Beijing 100190, China

**Keywords:** graphene/l-cysteine/gold electrode, glass carbon electrode, boron-doped diamond electrode, differential pulse stripping voltammetry, cadmium

## Abstract

Cadmium(II) is a common water pollutant with high toxicity. It is of significant importance for detecting aqueous contaminants accurately, as these contaminants are harmful to human health and environment. This paper describes the fabrication, characterization, and application of an environment-friendly graphene (Gr)/l-cysteine/gold electrode to detect trace levels of cadmium (Cd) by differential pulse stripping voltammetry (DPSV). The influence of hydrogen overflow was decreased and the current response was enhanced because the modified graphene extended the potential range of the electrode. The Gr/l-cysteine/gold electrode showed high electrochemical conductivity, producing a marked increase in anodic peak currents (*vs.* the glass carbon electrode (GCE) and boron-doped diamond (BDD) electrode). The calculated detection limits are 1.15, 0.30, and 1.42 µg/L, and the sensitivities go up to 0.18, 21.69, and 152.0 nA·mm^−2^·µg^−1^·L for, respectively, the BDD electrode, the GCE, and the Gr/l-cysteine/gold electrode. It was shown that the Gr/l-cysteine/gold-modified electrode is an effective means for obtaining highly selective and sensitive electrodes to detect trace levels of cadmium.

## 1. Introduction

Cadmium (Cd) has long been recognized as one of the most notable toxic heavy metals to ecosystems and human health [1,2]. It was used widely as an anticorrosive layer when electroplated onto steel, and cadmium compounds are used in batteries and as pigments. Cadmium and its compounds are of concern among main aqueous elements due to their high toxicity to aquatic organisms and their environments [3]. Accumulation of Cd can lead to severe chemical pneumonitis, lung cancer, and renal tubular dysfunction. Even the intake of trace levels of cadmium can lead to acute or chronic health issues such as kidney toxicity, cancer, and bone demineralization [4]. Therefore, the global urgency of on-site monitor, which can detect trace Cd(II) rapidly, sensitively, and reliably, has been increasingly underscored [5].

Conventional spectrometric measurements for metals include atomic absorption spectrophotometry (AAS) [6,7,8], inductively coupled plasma mass spectrometry (ICP-MS) [9,10], inductively coupled plasma atomic emission spectroscopy (ICP-AES) [11,12], atomic absorption spectrometry [13], and atomic fluorescence spectroscopy [14]. Complicated operations, costly instrumentation, and a long sample processing time limit the on-site application of these methods. A simple, low-cost, and user-friendly analytical method for on-site analysis is favored in cadmium detection. Electrochemical sensors provide promising approaches to monitor fresh water because of their simplicity and high sensitivity especially when differential pulse stripping voltammetry is applied. The traditional electrode for electrochemical detection of heavy metals has an imperfection that mercury is used and would bring about contamination [15]. Therefore, it is important to develop methods for environment-friendly efficient detection of Cd in aqueous solutions, especially at trace levels.

To address this issue, several nanoparticles have been modified on the surface of electrode based on nanotechnology, which has demonstrated exciting prospects in both chemical and optical determination of Cd(II) over the past decades. A variety of electrode surface modifications have been explored to increase sensitivity for metal detection. Sensitive, environment-friendly, and efficient determinations of heavy metal have been developed by grafting various kinds of nanomaterials onto the surface of electrodes. Platinum and gold electrodes in different forms and sizes have been widely used as backing material in electrochemical analysis due to their excellent electron transfer kinetics. However, they have been limited to detection in a positive potential range for most heavy metals due to the low hydrogen overvoltage that reduces the cathodic potential window, while the stripping peak of cadmium is comparatively negative. Platinum and gold electrodes can be combined with various nanoparticles and have a wide potential range.

Carbon-based electrodes, such as the glass carbon electrode (GCE), the boron-doped diamond (BDD) electrode, and the graphene-modified electrode, have a wide working potential range to make extensive analyses, low background currents to obtain high sensitivity, and stable structures to overcome harsh chemical environments. Graphene, a new type of environmental film, has also shown high hydrogen overpotential, which can suppress the hydrogen overflow on a gold electrode. Graphene is a 2D nanomaterial of sp^2^-bonded carbon atoms [16,17]. Moreover, graphene can be activated or connected by covalent bonding with glass carbon electrodes, and the modified electrodes retain graphene physical properties and chemical tenability including a high surface-to-volume ratio, a large active surface area, and an exceptional electrical property [18,19]. Since graphene formed by chemical activation possesses high electron transfer promoting ability [20] and excellent catalytic behavior, it has been widely used in novel chemical sensors on glassy carbon electrodes (GCEs) [21,22,23,24,25].

In this paper, graphene was modified on a gold electrode via a self-assembled method based on thiols and related molecules, which provided a way to perform Cd detection in a negative potential range [16]. We tried to combine carboxylic graphene (GN–COOH) with a gold electrode via thiol compound covalent bonding due to the function group hydroxyl (–OH) in the basal plane of carboxylic graphene and the amino-group in the gold electrode [26,27]. l-cysteine was selected as the linker between graphene and gold microelectrode as it provides fast electron transfer for electrochemical detection and amino group that can potentially join with graphene by dehydration-condensation reaction. The boron-doped diamond (BDD) electrode and the glass carbon electrode (GCE) were also examined for Cd measurements, and the results were compared with that of the Gr/l-cys/Au electrode.

## 2. Materials and Methods

### 2.1. Apparatus and Reagents

The purities of all reagents were in analytical grade except those stated separately. Double distilled water (18 MΩ·cm) was used to prepare for samples. Cadmium standard stock solution (100 mg/L Cd^2+^ in 3% nitric acid) was purchased from the China National Research Centre for Certified Reference Material. Cadmium samples were carefully diluted from the stock solution with double-distilled water. Carboxyl graphene (2 mg/L) was from Nanjing XFNANO Materials Tech Co., Ltd. (Nanjing, China). Dichloroethane (EDC) and *N*-Hydroxysuccinimide (NHS) were purchased from Sigma-Aldrich (St. Louis, MO, USA). Sodium acetate and glacial acetic acid were obtained from Xilong Tech Co., Ltd. (Shantou, China). Ferricyanide solution (0.5 mM) was added into 0.1 M KCl solution.

Electrochemical tests were conducted by Gamry Reference 600 electrochemical workstation (Gamry Instruments Co., Ltd., Warminster, PA, USA) using a three-electrode system based on a microelectrode chip. For comparison, traditional electrochemical bulk electrodes were used, while the glassy carbon electrode and the boron-doped diamond electrode (3 mm in diameter) served as the working electrode, the platinum disk electrode (3 mm in diameter) served as a counter electrode, and Ag/AgCl (saturated KCl) served as the reference electrode.

Field-emission scanning electron microscope (FE-SEM; S-4800) produced by Hitachi (Hitachi, Japan) was used to check the morphologies of the graphene.

### 2.2. Fabrication and Modification of Microelectrode Chip

**Figure 1:**
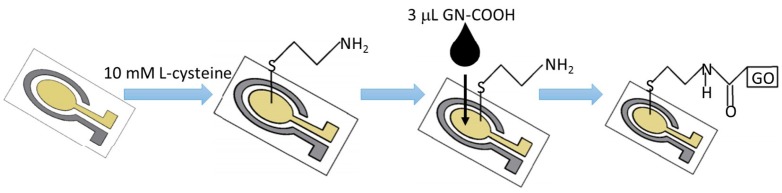
Schematic drawing of the Gr/l-cysteine nanocomposite self-assembly process.

The ring-disk microelectrode chip contains a gold working microelectrode, a platinium counter electrode. The microelectrode was prepared by the MEMS technique. The gold-working electrode was fabricated by standard optical lithography and a lift-off process with a diameter of 3 mm. Before modification, the gold microelectrode was cleaned in 0.01 M H_2_SO_4_ until a stable curve was obtained. The gold microelectrode was dipped in 20 µL of 10 mM l-cysteine (C_2_H_7_NS) solution for 6 h at 4 °C. The l-cysteine-modified gold electrode was obtained after 6 h of immersion. The modified gold microelectrode was washed with deionized water and dried in air for 24 h. Then, a mixed solution of NHS and EDC was applied into the carboxyl graphene (GN–COOH) solution for 15 min at 4 °C. Three microliters of the graphene-mixed solution was dispensed on the surface of the l-cysteine-modified gold microelectrode for 12 h at 25 °C. The hydroxyl group of the GN–COOH bond with the amino group of l-cysteine on the Au microelectrode with those steps. Finally, the carboxyl graphene (GN–COOH)-modified gold microelectrode was modified with l-cysteine as shown in Figure 1.

### 2.3. Electrochemical Methods of Differential Pulse Stripping Voltammetry (DPSV)

Differential pulse stripping voltammetry (DPSV) was applied to detect trace levels of cadmium under an optimized condition. All solutions were stored in a 20-mL Teflon bottle. DPSV parameters were optimized such that cadmium was deposited for 360 s at −1.1 V in 0.1 M acetate buffer (pH 4.50). The DPSV of electrodeposit metal was performed in the potential range of −1.1 to 0.5 V. The samples were homogeneous medium via stirring during the deposition steps, and the magnetic stirrer turned off for 120 s to equipoise prior to the DPSV scan.

## 3. Discussion

### 3.1. Characterization of Microelectrode Chip

**Figure 2:**
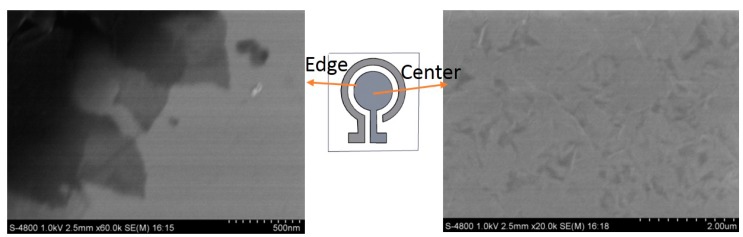
SEM of the center and edge of Gr/l-cysteine/Au electrode.

**Figure 3:**
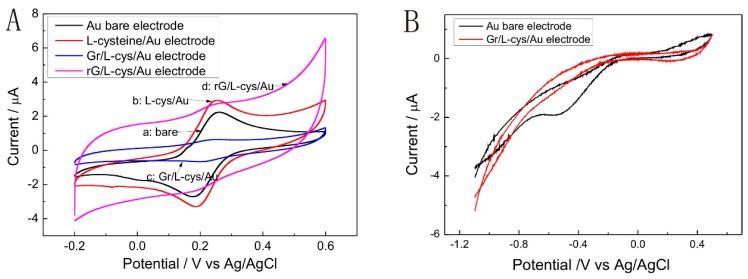
(**A**) Cyclic voltammograms of bare Au, l-cysteine/Au, Gr/l-cys/Au, and rG/l-cys/Au electrode in solution containing 0.5 mM ferri/ferrocyanide and 0.1 M KCl. (**B**) Cyclic voltammograms between −1.1–0.5 V of H_2_SO_4_, pH 4.50, at an Au bare electrode and Gr/l-cys/Au electrode.

The typical SEM images of Gr/l-cysteine/Au electrode are shown in Figure 2. The morphology of the GN–COOH/l-cysteine composite is rougher and sheets of graphene are clear in the edge of electrode. There are some thin folds on the surface in the center of the electrode. The electrochemical properties of different gold electrodes were characterized by CV. Figure 3a shows the voltammetric response of bare Au, l-cys/Au, Gr/l-cys/Au, and Gr/l-cys/Au electrodes in solution containing 0.5 mM Fe(CN)_3_^3−^ and 0.1 M KCl. Compared with the CV curve of the bare gold electrode (curve a), an obviously increased redox current was obtained by the l-cysteine-modified gold electrode (curve b). After the chemical bond between GN–COOH and l-cysteine layer, the redox current of Gr/l-cysteine/Au electrode (curve c) decreases compared with the l-cysteine/Au electrode. It can be seen from the reduced redox current that electron transfer was inhibited by the graphene layer. Reduction of GN–COOH to rGN–COOH (curve d) increases the conductivity of the catalyst. The existence of rGN–COOH results in a stronger combination of cadmium with the modified electrode. The subsequent stripping of cadmium was therefore kinetically harder, and the stripping potential became more negative. However, this potential shift brings about fierce hydrogen evolution, which may damage the deposition layer. Consequently, the rG/l-cys/Au electrode cannot be applied to Cd detection.

The influence of oxygen on the voltammetric behaviors of the bare gold electrode and the Gr/l-cys/Au on the surface is shown in Figure 3b. The cyclic voltammogram (CV) shows one reduction peak on the bare gold electrode at −0.5 V. Generally, the reduction peak was a two-electron reduction of O_2_ to H_2_O_2_ at the gold surface depending on the pH of the medium, which easily occurred in acidic media [28]. It can be seen that no significant reduction peak occurred at Gr/l-cys/Au electrode. Such behavior was due to the fabrication of graphene on the gold electrode and the graphene-modified gold electrode can be used in cadmium determination.

### 3.2. Optimazation of Depositon Time and pH Value of Detection Solution

**Figure 4:**
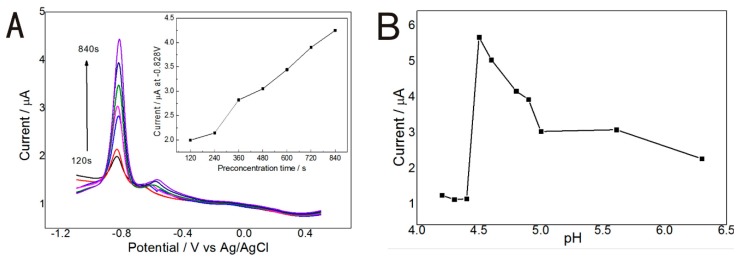
Effect of deposition time (**A**) on the differential pulse stripping voltammetry (DPSV) peak current of 10 µg/L Cd(II) at pH 4.50; Effect of pH (**B**) on the DPSV peak current of 10 µg/L Cd(II) with deposition time of 360 s.

In order to investigate the influence of the deposition time and pH value of the detection solution, experiments were designed with 10 µg/L Cd(II) by DPSV to identify the current response on the Gr/l-cys/Au electrode. As the pre-concentration time increased from 120 to 840 s, the peak currents for Cd^2+^ at pH 4.50 linearly increased, owing to the increased amount of cadmium ion on the Gr/l-cys/Au electrode. However, the stripping currents tended to slightly deviate from the linear region after 360 s, as shown in Figure 4A, due to the saturated loading of the electrode surface. The pH is another key parameter, which effects the sensitivity. Compared with the current responses at various pH values, the current response is obviously influenced by pH and reaches its highest value at pH 4.50 when the deposition time is 360 s (Figure 4B). For further study, pH 4.50 and 360 s deposition time were selected for Cd detection via the DPSV method.

### 3.3. Electrochemical Characterization of the Gr/l-Cysteine Composite-Modified Electrode

**Figure 5:**
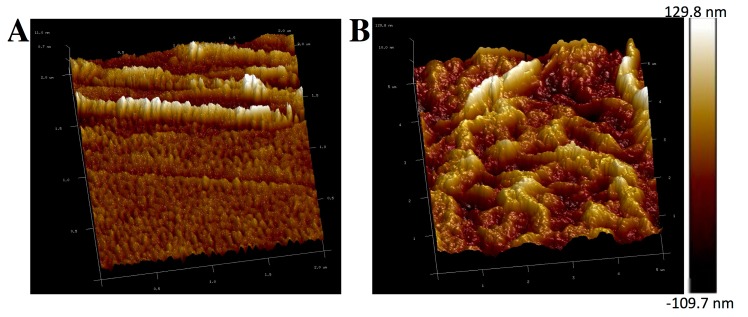
AFM images of bare (**A**) gold electrode and (**B**) Gr/l-cys/Au electrode.

The thickness of the one layer was measured by atomic force microscope (AFM). Figure 5 shows its surface morphology of the bare gold (A) and Gr/l-cys/Au (B) electrodes drawn by AFM. The height profile of one layer’s is about 120 nm along the line across the membrane surface. It is believed that 2D graphene sheets combined to create an interconnected framework. The overlap of graphene can greatly enhance the specific surface area of the Gr/l-cys/Au electrode and can give the unfolded graphene a significant character to capture metal ions during deposition time in aqueous phase [29].

**Figure 6:**
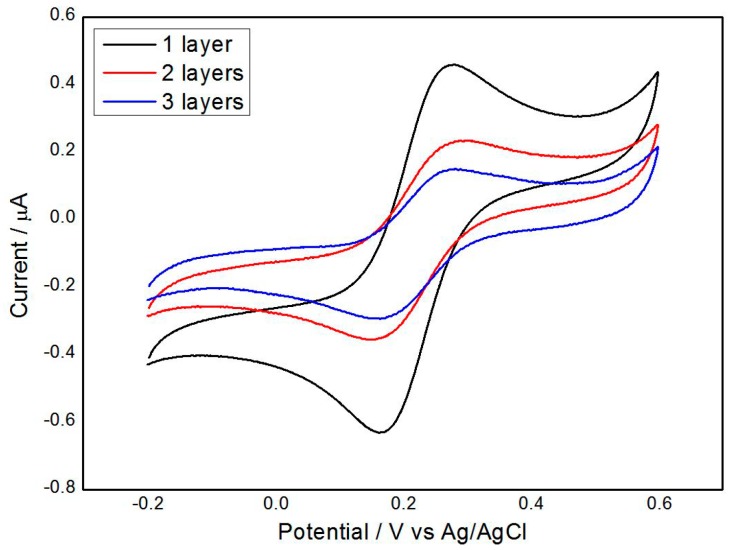
The representation of self-assembled graphene layers of the Gr/l-cysteine/gold microelectrode (all of the microelectrodes diameters were designed to yield 3 mm, and each graphene layer was modified by 3 µL of carboxylic graphene).

Figure 6 illustrates the representation of different self-assembled graphene layers. The number of layers means the times graphene drop-casting is performed. Graphene drop-casting comprises two steps: 3 µL of prepared GN–COOH solution was drop-casted onto the electrode surface; then, the electrode was air-dried for 12 h. As shown in Figure 6, the Gr/l-cysteine composite-modified electrodes generated improved the electrochemical character in a solution containing 0.5 mM ferri/ferrocyanide and 0.1 M KCl. The redox current decreased when the amount of GN–COOH loading increased from 3 to 9 µL, indicating that adding more GN–COOH to the electrode surface could diminish the electrochemical sensitivity of modified gold electrode. Graphene incorporation into l-cysteine is crucial for improving the electrochemical properties of the composite. One graphene layer displayed the best sensitivity, stability, and firm combination.

### 3.4. Analytical Performance of the Gr/l-Cys/Au Electrode, the GCE, and the BDD Electrode for Cd(II) Determination

**Figure 7:**
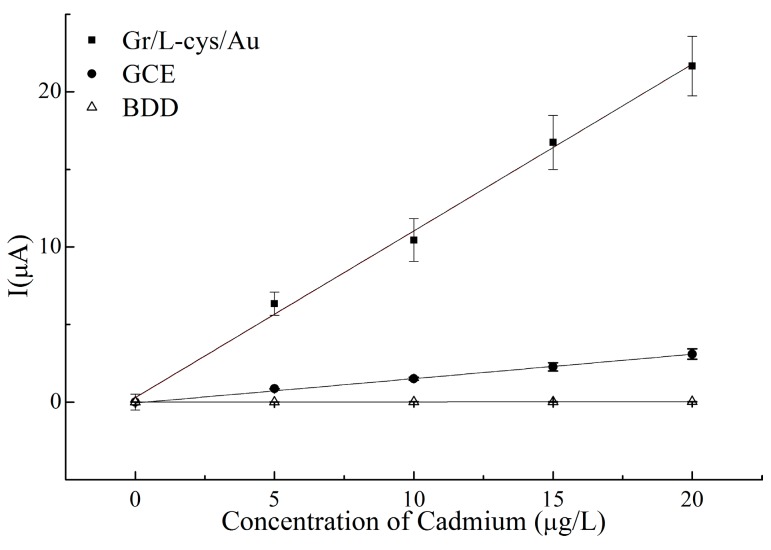
Representative calibration curves of the Gr/l-cys/Au electrode, the GCE, and the BDD electrode by DPSV for the determination of cadmium ions with corresponding error bars (*n* = 3).

To detect trace levels of Cd(II), DPSV is used because of its high sensitivity and linearity [30]. To increase electrode sensitivity, l-cysteine was used to connect the electrode surface with the graphene by a self-assembly process. Calibration samples of deionized water (DW) were used to test the analytical performance of our method. Samples were spiked with 0, 5, 10, 15, and 20 µg/L cadmium(II) from standard stock solutions. The resulting calibration plots are linear over a range from 5 to 20 µg/L, with correlation coefficients exceeding 0.95 (Figure 7). The analytical performance of all three electrodes for heavy metal detection (such as cadmium, lead (Pb)) was also compared with other carbon-based electrode reported previously, which were summarized in Table 1.

Furthermore, carbon-based electrodes have obtained significant attention in previous reports because of its environmental-friendly effect and high sensitivity, especially the GCE, the BDD electrode, and the graphene [31,32]. A variety of carbon-based electrodes have been experimented with to explore the detection sensitivity of heavy metals. The electrochemical performance of the Gr/l-cysteine nanocomposite-modified gold electrode was compared to the GCE and the BDD electrode in similar systems for measuring trace levels of Cd(II). Table 1 shows that good linearity is acquired within the 5–20 µg/L range. The limits of detection (LODs) are determined based on the signal-to-noise ratio at low levels (S/N = 3) and are 1.42, 0.3, and 1.15 µg/L for the Gr/l-cys/Au electrode, the glass carbon electrode, and the boron-doped diamond electrode. The sensitivities go up to 0.18, 21.69, and 152.0 nA·mm^−2^·μg^−1^·L for, respectively, the BDD electrode, the GCE, and the Gr/l-cys/Au electrode. The sensitivity of the Gr/l-cys/Au electrode was higher than the other carbon-based electrodes, and the detection limit of the proposed method was comparable to other electrochemical detection methods. It was thus shown that the Gr/l-cys/Au-modified electrode is an effective method for obtaining highly selective and sensitive electrodes for detecting trace levels of cadmium.

## 4. Conclusions

In this work, the gold disk microelectrodes with the sensing diameter of 3 mm were fabricated by the MEMS technique on a glass wafer. The novel Gr/l-cysteine film was successfully modified on gold microelectrode, and the structure of the microelectrode chip demonstrated the fabrication of this composite electrode. The Gr/l-cysteine/gold electrode showed microelectrode behavior and yielded a high, steady current. The electrochemical characteristics of the microelectrode during modifications were investigated by cyclic voltammetry in [34]. The representation of a different self-assembled graphene layer suggests that one graphene layer is very secure and achieves better sensitivity. The effects of deposition time and pH on the DPSV peak current were investigated. The calibration plots of the Gr/l-cysteine gold microelectrode, the GCE, and BDD electrode for Cd(II) determination are linear with a range from 5 to 20 µg/L, with correlation coefficients exceeding 0.95. The Gr/l-cysteine gold microelectrode showed the highest sensitivity compared with the bare glassy carbon and boron-doped diamond due to the use of graphene and microelectrodes. In addition to the present work, this modified microelectrode can also be used for the detection of other trace metals that have lower deposition potentials than cadmium.

## Figures and Tables

**Table 1:** Electrode performance for measuring heavy metals.

Electrode	Metal Ion	Sensitivity (nA·mm^−2^·μg^−1^·L)	Linearity Range (μg/L)	Correlation Coefficient	LOD (μg/L)	Reference
Crown/GCE	Cd(II)	16	7.9–191	0.99	2.4	[33]
MWNTs/GCE	Cd(II)	135	2.8–110	0.99	0.67	[34]
N@MOG-C/GCE	Cd(II)	89	2.8–55	0.99	0.25	[35]
b^2^SPE	Pb(II)	60	5–110	0.99	1.10	[36]
GCE	Cd(II)	21.69	5–20	0.998	0.30	This work
BDD	Cd(II)	0.18	5–20	0.950	1.15	This work
Gr/l-cys/Au	Cd(II)	152.0	5–20	0.997	1.42	This work

MWNTs: multi-wall carbon nanotubes; N@MOG-C: nitrogen-doped porous carbon material; b^2^SPE: back-to-back screen-printed electrode.

## Abbreviations

The following abbreviations are used in this manuscript:

Cd	cadmium
Gr	graphene
l-cys	l-cysteine
GCE	glass carbon electrode
BDD	boron-doped diamond
DPSV	differential pulse stripping voltammetry
